# A gender-responsive breeding approach to the intensification of sesame (*Sesamum indicum* L.) production in the Maradi region of Niger

**DOI:** 10.3389/fsoc.2024.1254094

**Published:** 2024-05-22

**Authors:** Sitou Lawali, Seyni Boureima, Salissou Idi

**Affiliations:** ^1^Unité Mixte de Recherche: Sustainable Development, Societies and Adaptation to Climate Change, Maradi, Niger; ^2^Faculty of Agronomy and Environmental Sciences, Dan Dicko Dankoulodo University, Maradi, Niger; ^3^FUOPAN Farmer's Federation of Maradi, Maradi, Niger

**Keywords:** sesame, value chain, gender, breeding, strategies, Niger

## Abstract

Climatic variability and a decrease in soil fertility have had a detrimental effect on the productivity of the main rainfed crops in Niger (millet, sorghum, and cowpea) and led to a deterioration of the nutritional status and income of the country's farmers. The spatio-temporal variability in rainfall has led rural populations to diversify their farms by integrating sesame (*Sesamum indicum* L.) into their cropping systems because of its low water and fertilizer requirements. Sesame is increasingly becoming a significant source of income for farmers, and it contributes to their food and nutritional security. To boost the production of sesame and facilitate its rapid adoption, our breeding program focused on participatory breeding and varietal selection with the inclusion of gender-specific preferences, from the variety design to the evaluation of new lines on farms. This case study shows that, although women have more experience in sesame cultivation than men, they have less access to production factors such as land. This limited access is especially problematic, as recent trends in land tenure mean that the poorest are no longer able to exploit large areas of cultivable land. It also evidenced that the varietal preferences of sesame growers as well as the mastery of production techniques are a function of the livelihoods and the investment capacity of actors in the value chain. Our study found that men mainly prefer production traits, whereas women have fewer trait preferences, and their preferences tend to be related to marketing and processing. This finding highlights the contrasting roles and responsibilities between men and women in the sesame value chain. Therefore, the inclusion of complementary traits preferred by women and men, provided that they are not negatively correlated with a variety profile, will help meet the full range of needs across the value chain. We recommend the inclusion of gender research in setting breeding goals prior to variety design.

## Introduction

Sesame (*Sesamum indicum* L.) is an annual oilseed crop belonging to the *Pedalliaceae* family. It is cultivated for its seeds, which are rich in oil (50%), protein (23%), vitamins (such as E and B), and amino acids (methionine, cystine, arginine, and leucine). In West Africa, sesame is cultivated across approximately 1,417,115 ha for an annual production of 941,021 tons (FAO, 2022) in pure stands or in association with cereals (e.g., millet and sorghum).

In Niger, sesame is traditionally an underutilized crop that was produced exclusively by women for self-consumption within the household, often in the form of condiments. Women therefore have a great deal of responsibility in the selection and maintenance of traditional varieties and in decisions on the uses of harvested seed. In recent decades, the demand for sesame seed has risen sharply on the international market, transforming the plant from a neglected crop to a high-value commercial crop. More than 5,600 products derived from sesame have been developed by the processing industry in China (Zhang, 2019). This renewed interest in sesame seeds on the international market has attracted more men to sesame cultivation across large areas. Since men have greater access to production factors (e.g., land and finance) than women, production has predominantly become the work of men, while women, who were once the main producers, have been relegated to the processing and marketing of processed products. Men mainly sell sesame seeds to women on the local market or export the crop to neighboring countries. As such, the role of women has transitioned from that of producers to become the main local processors of sesame seeds.

This transformation is taking place in the context of global climate change and an expanding population that is putting high pressure on the already degraded agricultural lands. It is therefore important for the development of the sesame sector that varietal creation programs take into account for the preferences of all actors involved in the value chain, without forgetting the biotic and abiotic factors dictated by the changing climate.

Until recently, sesame breeding programs have focused on a limited number of traits such as yield, drought tolerance (Diouf et al., 2010; Boureima and Van Damme, 2012; Boureima et al., 2016), and biotic stress. Our limited knowledge of the preferred traits of value chain actors comes from lessons learned during participatory varietal selection (PVS) programs on other crops. Not only are those studies not sesame-specific but they were also conducted in the later stages of the varietal selection process and thus missed opportunities for targeted crop improvement.

In general, and in Niger, in particular, sesame is an orphan crop in that no international agricultural research center has a research mandate for sesame. Consequently, sesame has attracted only limited scientific research to date. Thus, lessons from other crop breeding efforts that used PVS have been an entry point to help us understand the differences that exist between men and women with respect to the preferred agronomic traits of sesame varieties.

Recently bred lines and introduced accessions were evaluated by our team in multi-location trials in farmers' fields by both researchers and sesame farmers (women and men) at all stages of crop development. This study revealed that some traits, such as white seed coat color, were specifically preferred by women for their oil quality. However, our motivation to pay more attention to gender differences in trait preferences derived from the participation of our team (the sesame breeder and the social scientist) in a training course offered by the organization Gender-Responsive Researchers Equipped for Agricultural Transformation (GREAT) in 2021. This experience fundamentally changed the way we approach selection objectives by integrating gendered preferences into the product profile.

The main purpose of this study was to share experiences in terms of the changes induced on the actors involved in a participatory and inclusive selection program while favoring equity in decision-making.

## Methods

### Contribution to the field statement

The sesame breeding program involves one breeder, one social scientist, and MS and PhD students in the Faculty of Agronomy and Environmental Sciences at the Dan Dicko Dankoulodo University of Maradi, Ali Dan Sofo, Niger. They collaborated with members of two farmers' organizations (FUOPAN SAA in Maradi and FUBI in Zinder region) and one female processors' organization in Tessaoua, in the Maradi region. This collaboration could be related to the selection of participants, and if so, it not only describes but more importantly shows that user representatives have been part of the breeding program. In much of the literature, direct representatives of users have often been left out, and representation of users has been left to social scientists and marketing specialists. Cavicchioli et al. (2023) found that many breeders get concrete feedback directly from users, and those users are even directly part of product advancement, although often not formally recognized. The target beneficiaries are smallholder farmers (both women and men) and processors (predominantly women), as well as traders and the scientific community at national and international levels.

### A gender-responsive approach to breeding

In most breeding schemes, the primary goal is to improve the yield of the variety selected to the point that only traits that are directly related to yield are of importance to the breeder. However, since a given crop can be intended for different users and markets, it is important to consider the diversity of end uses for any product that the breeding program produces. We brought together a multidisciplinary and interdisciplinary team to respond to the concerns of the various stakeholders in Niger's sesame sector. In this study, we describe the use of a value chain approach to optimize our sesame breeding program.

In 2012, the predominant belief was that shattering, i.e., the loss of seeds by the opening of the capsules at maturity, was the major constraint to sesame production. In response, a non-shattering sesame line was developed (Boureima, 2012), but this variety was not adopted by both male and female producers for the sole reason that women, who were primarily responsible for the threshing of the capsules, struggled to recover seeds from the capsules because the seeds were tightly retained in the placenta. In contrast, seeds from shattering varieties could simply be recovered by inverting the dried plant so that seeds could flow freely from the capsules.

After the failed adoption of this non-shattering variety, and by taking into account the preferences of women as threshers of the capsules as well as the preferences of sesame producers (predominantly men), we reoriented our breeding program. Shattering at maturity can cause production losses in the order of 70–80% (Boureima, 2012) and therefore must be considered in the creation of new varieties. We sought to not only produce varieties that are resistant to shattering without being indehiscent to improve sesame yields but also facilitate the threshing of the capsules at harvest by women.

This experience was the trigger to introduce gender-specific preferences to our breeding objectives at all levels, including the methods for evaluating new material on-station or on-farm, variety design, priority traits for the breeding program, criteria to use for evaluating the importance of different traits, and the choice of lines to be advanced to the following generations. Since 2021, our breeding strategy has focused on the development of white sesame seed varieties to satisfy the world market, but these varieties must also display an appropriate level of shattering resistance to avoid seed loss at maturity while still facilitating threshing by women. Our lessons learned in sesame breeding will be broadly applicable because threshing is exclusively done by women across various crops in Niger.

Gender considerations were applied to all methods used in this study and involved all stages of the research (Table 1). This approach allows for consideration of social relations as well as the specific preferences of women and men. We aim to develop the sesame value chain while offering the same opportunities to all stakeholders to be rewarded for their efforts.

**Table 1:** All activities of the sesame breeding program currently incorporate gender-specific considerations.

**Activity**	**Description of work**
Participatory varietal selection (PVS)	In this study, an evaluation was conducted on village demonstration plots in the communes of Tessaoua and Bandé. The aim was to evaluate selected varieties according to gender-specific trait preferences throughout the selection process and according to the criteria defined by the categories of stakeholders.
Experimenting farmers formally organized in groups or committees or networks to contribute to the breeding program	The experiments were conducted according to a participatory approach through field trials and operations in the form of field schools, farmer exchanges, and a validation workshop for the consequent results. Two farmers' organizations were involved in these experiments.
Social survey research	We conducted social survey research for descriptive statistics on household characteristics (age, gender, education, ownership, partition of agricultural labor, etc.).
Value chain analysis and mapping	The study considered the links in the value chain, mainly selection, production, processing, and marketing. The objective was to understand the role of the different value chain participants, both men and women.
Study of trait preferences	In 2022, for the first time, we conducted a survey on the sesame value chain in the Maradi and Zinder regions to understand the trait preferences of both men and women and to identify gender-related constraints to production factors. The study has a double objective: the first is to understand the preferences of sesame seed producers and processors and to use statistical models to compare and assess preferences by gender. The second objective is to elucidate whether men and women have equitable access to inputs as driving forces to adopt a newly released sesame variety.
Farmer-to-farmer visits and exchanges	Visits to the experiment sites by farmers have the advantage of strengthening their capacity and motivation to adopt novel technologies.
Farmer field school experiments and demonstrations	Farmers' field schools were set up in the villages of Gounaka, Maiguizaoua, and Dadin Sarki. These allowed pilot farmers, including smallholder farmers (men and women), to adopt and master the technical processes of sesame production and for additional farmers to gain interest in the crop.

The information used in this case study was obtained from two recent studies (Yaou, 2017; Boureima and Yaou, 2019) conducted on station and on the farmers' land with federations of farmers' organizations in two regions of Niger, namely, Maradi and Zinder. The first approach was based on participatory varietal selection conducted from 2016 to 2018, through which 10 sesame varieties were evaluated with the participation of groups of male and female producers at five locations in the Maradi and Zinder regions. The varieties were tested in randomized, complete blocks with four replications at each location. Producers, including men and women, were organized into groups belonging to the federations of farmers' organizations, FUOPAN SAA in Maradi and FUBI in Zinder and were involved in all cultivation operations. The balance between men and women was not taken into account in this study. In addition, the team worked with the farmers' organizations to arrange group visits to assess the attributes of the varieties according to their stage of development (flowering, fruiting, general appearance of the plant, and maturity). Each time, we collate the growers' impressions by asking them to place a branch of a shrub on the most preferred variety across the four repetitions. The parameters monitored by the pilot farmers were the earliness of emergence, the variety that reacted more to the application of fertilizer, comparison between local and improved varieties for agronomic traits, flowering time and duration, attacks by insects and diseases, lodging susceptibility of different varieties, the number of capsules, seed size, the best yield, and the preferred seed color of the varieties.

These exercises were repeated throughout the development cycle and allowed us to form a synthetic compilation of which characteristics were preferred by the producers. During this period, we only focused on the farmers' overall preferred characteristics, regardless of gender. The sole aim was to describe an ideotype variety for a sesame farmer. The selection of participants was done by a focus group with farmers' organizations based on the farmers' experience in sesame cultivation. The *t*-test and chi-square test were carried out on the data collected for continuous and categorical variables, respectively.

In 2021, following the failed attempt to introduce a non-shattering variety that did not meet the expectations of women, and in response to input from GREAT, we reassessed and adopted a new approach based on the inclusion of gender preferences across the entire selection process from the design of the variety to the monitoring and evaluation of the germplasm developed through the PVS. The inclusion of female processors in the evaluation stage was the main shift in the breeding strategy since their knowledge and preferences will have an outsized impact on the sesame value chain. Farmer field schools were conducted across the five locations for demonstration tests and to share the performance of the new lines compared to local sesame varieties.

## Results

The male and female producers who were involved in this pilot experiment experienced a modern approach to sesame breeding, including the participation and inclusion of both women and men in decision-making to describe an ideal improved sesame variety. In terms of plant emergence, farmers' observations indicate that the varieties with the highest emergence were the most preferred by both men and women. At the maturity stage, highly branched varieties with a higher number of capsules were more appreciated by both men and women. With respect to fertilizer response, the producers preferred the varieties that were most responsive to fertilizer, irrespective of their gender. Resistance to lodging at the reproductive phase is another characteristic that would be expected for an improved variety. The male producers preferred varieties with larger seeds and sand capsules; however, after threshing the capsules, the white-colored variety was most preferred by the female producers. Details of this study are available in Boureima et al. (2017).

In summary, this participatory evaluation of varieties demonstrated that earliness, number of capsules per plant, large capsules, high branching, and delayed shattering are the major traits preferred by men, whereas the trait preferred by women was the white color of sesame seeds. This information is of great value for us to set our new breeding objectives in the sesame program and highlights the fact that men and women may even have conflicting interests in crop traits.

The second study was a diagnostic survey of 580 rural sesame-producing households in the Maradi region using a structured questionnaire on the varietal preferences of producers (men and women) and processors (women) as well as constraints related to access to production factors. This study was conducted from July to October 2022 by the social scientist and one master's student (for his thesis research) in collaboration with the breeder, and the socioeconomic characteristics of the respondents are given in Table 2. This questionnaire took up a variety of characteristics that were preferred by farmers and processors.

**Table 2:** The chi-square test on socioeconomic characteristics of respondents to the diagnostic survey of rural sesame-producing households.

	**Continuous variables**				
	**Men**		**Women**		
**Characteristics**	**Mean**	**SD**	**Mean**	**SD**	***t*** **-test**
Age (years)	48.20	12.59	50.82	11.38	*t* = 0.94; *p* = 0.34
Household size	13.51	6.36	10.68	8.58	*t* = −1.94; *p* = 0.05
Land size in ha	5.14	4.15	3.18	3.35	*t* = −2.15; *p* = 0.03
Sesame land in ha	1.62	1.28	1.23	0.53	*t* = −1.41; *p* = 0.15
Sesame experience (years)	10.36	6.05	14.27	9.21	*t* = 2.77; *p* = 0.005
**Categorical variables**			
	**Men**	**Women**	***p***
Inheritance	96.12	45.45	^***^
Rental	3.87	9.10	ns
Gift	0.00	0.00	ns
Purchase	43.8	22.72	^*^
Renting	3.48	4.54	ns
Pledge	8.90	0.00	ns

Significance is indicated at 5% (^*^) and 0.1% (^***^).

The average age was 48 years for men and 51 years for women (Table 2). The number of dependents varied from 14 among households headed by men to 11 among households headed by women. The questionnaire confirmed that women have more experience in sesame cultivation (14 years on average) than men (10 years on average). For production factors such as land, the results showed that, although women and men cultivate similar areas of the sesame crop, women have less access to smaller land areas (3.18 ha) than men (5.14 ha), while farming remains the main activity for all respondents, regardless of gender.

The two major modes of land tenure in the study area are inheritance and purchase. However, according to the results of the analysis, there is significant gender discrimination with regard to these modes of land tenure. Men have significantly more access to land through inheritance (96.12%) and purchase (43.8%) compared to women (45.45 and 22.72%, respectively). The alternatives for land tenure are renting, giving, and pledging, which are less frequently practiced but have no significant discrimination based on gender.

### Analysis of preferences by gender

The results of the survey revealed that, according to the three models of trait preferences, i.e., identical, separate (or totally discrete), and overlapping, the preferences of women and men are not always identical (Table 3). Men mostly prefer traits that relate to agronomic performance, while women are much more interested in traits that relate to processing. Some traits overlap, such as red varieties, delayed dehiscence of capsules, and number of branches, meaning that even if some preferences are not identical, they are still reported by both men and women. Thus, a breeding program must provide compromise and incorporate the specific needs of women to complement those of men in the variety design to develop an acceptable variety profile. To achieve this goal, we focused on shattering resistance and white seed color while maintaining resistance to lodging. According to the respondents, four sesame derivatives are produced by women in the department of Tessaoua. These are sesame oil, sesame paste used as condiments for cooking, sweet sesame biscuits, and salty sesame biscuits. All these products are sold at the local markets by women and mostly by young girls. The roasted sesame seeds are generally used for home consumption. The money from these activities is usually used by women to support themselves and their families during the lean season. In this study, women's and men's preferences are motivated by the specific task and role they have come to play. They are a function of the livelihoods and investment capacity of actors in the value chain. The sesame value chain creates opportunities for value addition and employment in processing and marketing in rural areas, especially for women.

**Table 3:** Synthesis of the case study according to the three trait preference models for some major traits.

**Identical preferences**	**Separate preferences**		**Overlap**
	**Men**	**Women**	**Red seeds**
Yield	Resistance to lodging	High oil content	Delayed dehiscence
Earliness	Several capsules per plant	White seed coat	Several branches
	Resistance to diseases		
	Tolerance of drought		

## Discussion

### Good practices

By recognizing the importance of gender preferences and prioritizing gender-responsiveness in the breeding program, it has been possible to highlight preferred traits according to the categories of stakeholders throughout the value chain. The success of a breeding program can be evaluated on the basis of public or private utility and, therefore, the feedback from the users of the new variety. This case study demonstrates the importance of a multidisciplinary and interdisciplinary approach to establishing a breeding program that meets the expectations of all beneficiaries. The key elements of this approach include the following.

*1. Information about gender is taken into account in decisions about which market(s) or end users the breeding program will target*.

The need for gender-disaggregated data provides new, complementary, and actionable information for setting breeding objectives. There is a real need for research dedicated to understanding gender preferences in agriculture. The importance of gender considerations in crop improvement programs has been emphasized in numerous studies (World Bank et al., 2009; Galiè et al., 2017). In our breeding program, a budget for gender will be standard as it is essential for the inclusion of gender in the program to help set our breeding objectives.

Although some traits overlap in terms of preferences between men and women, for some products, there is a clear difference in choice or preference according to gender. Women are mainly processors, but some of them have their own sesame field. In contrast, all men are producers and sellers. Taking these differences into account will make it possible to exploit complementarities to develop a product that is widely appreciated by a full range of stakeholders and end users. However, a related challenge has emerged around goals and expectations. Overall, the focus is on identifying trait preferences. To be of practical value for breeding, a preference should be linked to a usable trait, i.e., specific, quantifiable, and heritable traits that are technically feasible for the breeder to target. Some identified preferences, while still relevant to customers, are not actionable by breeding programs, for example, a high number of capsules and lodging resistance. Varieties with a high number of capsules are susceptible to lodging because of the weight of the capsules.

Gender-specific trait preferences are most useful if used early in the program to help envision a target varietal design and then define breeding objectives. Participatory evaluations with the inclusion of gender specialists, producers, and processors can better refine the selection and achieve a set objective that will increase the chances of adoption of new varieties.

This finding ensures good collaboration between all participants seeking to improve the wellbeing of the population. A gender-based approach, in which producers and processors are the key focal points of all varietal selection, has the merit of establishing mutual trust between researchers and end-users and facilitating the ultimate adoption of improved varieties. The participatory approach developed in this program has enhanced the approach of researchers who currently consider the socioeconomic context, needs, and conditions for accessing resources in their decision-making.

We encourage gender mainstreaming in our sesame breeding program to promote gender equality and the empowerment of women. This consideration is introduced early in the breeding process.

The demonstration plots were managed by pilot producers (women and men) at five locations who have promoted the integration of vulnerable groups (particularly women). All the achievements have been developed in a collaborative way, contributing to solidarity among community members, which consequently strengthened social cohesion. In accordance with the objectives set by the research team, the availability of improved varieties to producers is expected to influence an extension of the cultivated area and the productivity of sesame in Niger. We have emphasized the training of producers, both men and women, and the inclusion of gender responsiveness for a more efficient and effective evaluation of candidate varieties.

*2. Information about gender is taken into account in decisions about what types of farmers should participate in evaluations*.

From the PVS experiment, it is evident that a field evaluation of varieties that is undertaken without women is an incomplete study because it ignores a significant portion of the end users, which is likely to reduce the adoption rate and the desired impact of the variety.

In the sesame breeding program, important changes have been observed among both producers and researchers as a result of the participatory and inclusive approach developed through the program's activities. All research questions are formulated according to the real concerns of the stakeholders. The program brings together plant breeders, socioeconomists, environmentalists, agricultural advisory support technicians, farmers' organizations, and students. In this approach, the research protocol is co-constructed and implemented in a real environment using the “field school” concept, with researchers, growers, and students. The main stages involved a participatory gender approach, the results of which provide guidelines for variety development based on stakeholder preferences, followed by a participatory on-site evaluation with growers. Feedback on the results of the participatory approach is provided through an inclusive workshop that includes all actors. This is, for example, comparable to the methodology followed by Forsythe et al. (2021), whose implementation is reflected in the study of Forsythe et al. (2024): The transdisciplinary team worked together from the beginning, participating in activities and exchanging information and carrying out research together regardless of what the dominant discipline was in each activity. This collaboration meant that, apart from having people from the relevant different disciplines involved, which is not a guarantee for success, people actually engage in a meaningful way and on the same level (Page, 2010). This engagement means that participation is also performative and not only deliberative (Richards et al., 2010). It is through the effervescence in a (task) group, created by the act of performing similar activities, that institutions and epistemologies are created (McFeat and Belshaw, 1974; Collins, 2004; Perri 6, 2007) that can be stronger than the interest of the larger institutions that each of the individual people from the different disciplines and backgrounds are part of given their disciplinary schooling, affiliation, and experience. In the case of the sesame breeding program, this could state that this has avoided issues of power dynamics often prevalent in multidisciplinary decision-making (Tarjem I., 2022; Tarjem I. A., 2022; Cullen et al., 2023). This experience has fundamentally changed the way researchers set research objectives in the sesame breeding program by integrating the gender preferences of stakeholders. The participatory approach developed has had the merit of influencing a change in the behavior of researchers, who currently take into account the socioeconomic conditions of stakeholders and their preferences. The approach also enabled researchers, producers, extension agents, and students to work together as equals to achieve research objectives. Finally, it strengthened the strong social relationships between sesame value chain actors.

### Summary and lessons learned

This approach to integrating gender responsiveness into the breeding program has yielded the following key reflections and outcomes.

Identification of the specific needs of stakeholders is crucial and should be applied to breeding objectives.

Plant breeding can be planned through a gender-responsive lens to promote the active involvement of farmers at all stages of the breeding program.

Inclusion and consideration of women's preferences are of utmost importance for a breeding program.

Gender mainstreaming can be applied as a strategy for integrating gender concerns into the analysis, formulation, and monitoring of policies, programs, and projects.

Gender inclusion in our breeding program is likely to result in the broad adoption of our newly released varieties.

The development of a win-win relationship between men and women improves socioeconomic development and community resilience strategies.

Implemented on a small scale, the gender-based sesame breeding program in the Maradi region has had a striking effect in terms of supporting community resilience in general and the resilience of the socially disadvantaged groups in particular.

A multidisciplinary team, including breeders and social scientists, is advantageous for a breeding program and can lead to novel solutions.

For those starting out a breeding program, we recommend a team approach to ensure efficient collaboration, with both men and women working together to set breeding objectives.

Further research on gender-responsive breeding is recommended to facilitate greater, more inclusive benefits from new varieties, which could boost prosperity and thereby reduce the migration of women and youth to escape poverty and food insecurity. To favor the adoption of new crop varieties, the breeder needs to ensure these varieties match the target use contexts, which is particularly challenging due to the heterogeneity of socioeconomic and environmental factors among smallholder farmers. For these multidimensional aspects of new variety development, the breeder needs to cooperate with other domains, such as social sciences, to design his breeding pipeline.

## Data availability statement

The raw data supporting the conclusions of this article will be made available by the authors, without undue reservation.

## Ethics statement

Ethical approval was not required for the study involving human participants in accordance with the local legislation and institutional requirements. Written informed consent to participate in this study was not required from the participants in accordance with the national legislation and the institutional requirements.

## Author contributions

SL: Conceptualization, Formal analysis, Investigation, Methodology, Supervision, Writing – original draft. SB: Conceptualization, Data curation, Formal analysis, Investigation, Methodology, Writing – original draft. IS: Formal analysis, Investigation, Methodology, Supervision, Writing – original draft.
